# Snowball 2.0: Generic Material Data Parser for ChemDataExtractor

**DOI:** 10.1021/acs.jcim.3c01281

**Published:** 2023-11-07

**Authors:** Qingyang Dong, Jacqueline M. Cole

**Affiliations:** † Cavendish Laboratory, Department of Physics, University of Cambridge, Cambridge CB3 0HE, U.K.; ‡ ISIS Neutron and Muon Source, STFC Rutherford Appleton Laboratory, Harwell Science and Innovation Campus, Didcot, Oxfordshire OX11 0QX, U.K.

## Abstract

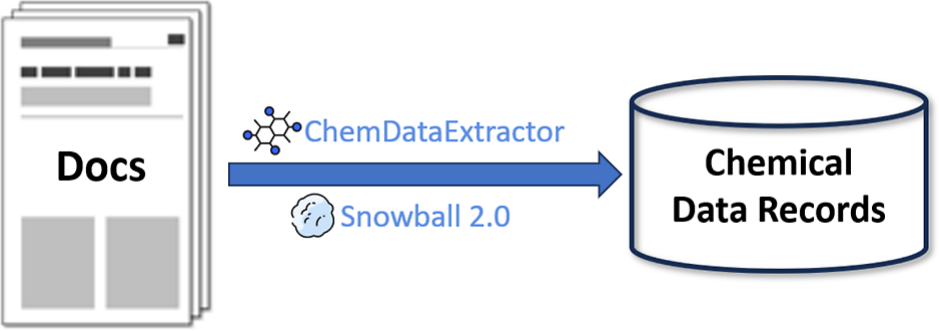

The ever-growing amount of chemical data found in the scientific literature has led to the emergence of data-driven materials discovery. The first step in the pipeline, to automatically extract chemical information from plain text, has been driven by the development of software toolkits such as ChemDataExtractor. Such data extraction processes have created a demand for parsers that efficiently enable text mining. Here, we present Snowball 2.0, a sentence parser based on a semisupervised machine-learning algorithm. It can be used to extract any chemical property without additional training. We validate its precision, recall, and *F*-score by training and testing a model with sentences of semiconductor band gap information curated from journal articles. Snowball 2.0 builds on two previously developed Snowball algorithms. Evaluation of Snowball 2.0 shows a 15–20% increase in recall with marginally reduced precision over the previous version which has been incorporated into ChemDataExtractor 2.0, giving Snowball 2.0 better performance in most configurations. Snowball 2.0 offers more and better parsing options for ChemDataExtractor, and it is more capable in the pipeline of automated data extraction. Snowball 2.0 also features better generalizability, performance, learning efficiencies, and user-friendliness.

## Introduction

With the volume of material-related research growing at increasing rates, it is possible to predict new material information by analyzing existing chemical data via various computational methods. Thus, data-driven materials discovery has been motivated as a faster alternative to conventional materials research,^1−4^ which is largely realized by “trial-and-error” experimentation and serendipity. Large-scale projects such as the Materials Genome Initiative^5^ and the Materials Project,^6^ alongside other computationally focused projects like AFLOW,^7−9^ have given rise to numerous open-source databases of chemicals and advancements in data science, both of which are crucial to scientific and industrial innovation.

The success of data-driven material research relies on having available large sets of chemical data, from which patterns of structure–property information can be inferred, and new property information can be predicted by machine-learning algorithms. So naturally, the first step is to extract chemical data from scientific literature via natural language processing (NLP) techniques. While data extraction from tables and specific sections of scientific papers can be achieved via software with high accuracy and automaticity,^10−15^ processing raw text has proven to be more difficult since chemical information is scattered across paragraphs of papers in highly fragmented and unstructured forms. Hence, sentence parsing has become a priority in text mining for the automated generation of chemical databases.

**Figure 1:**
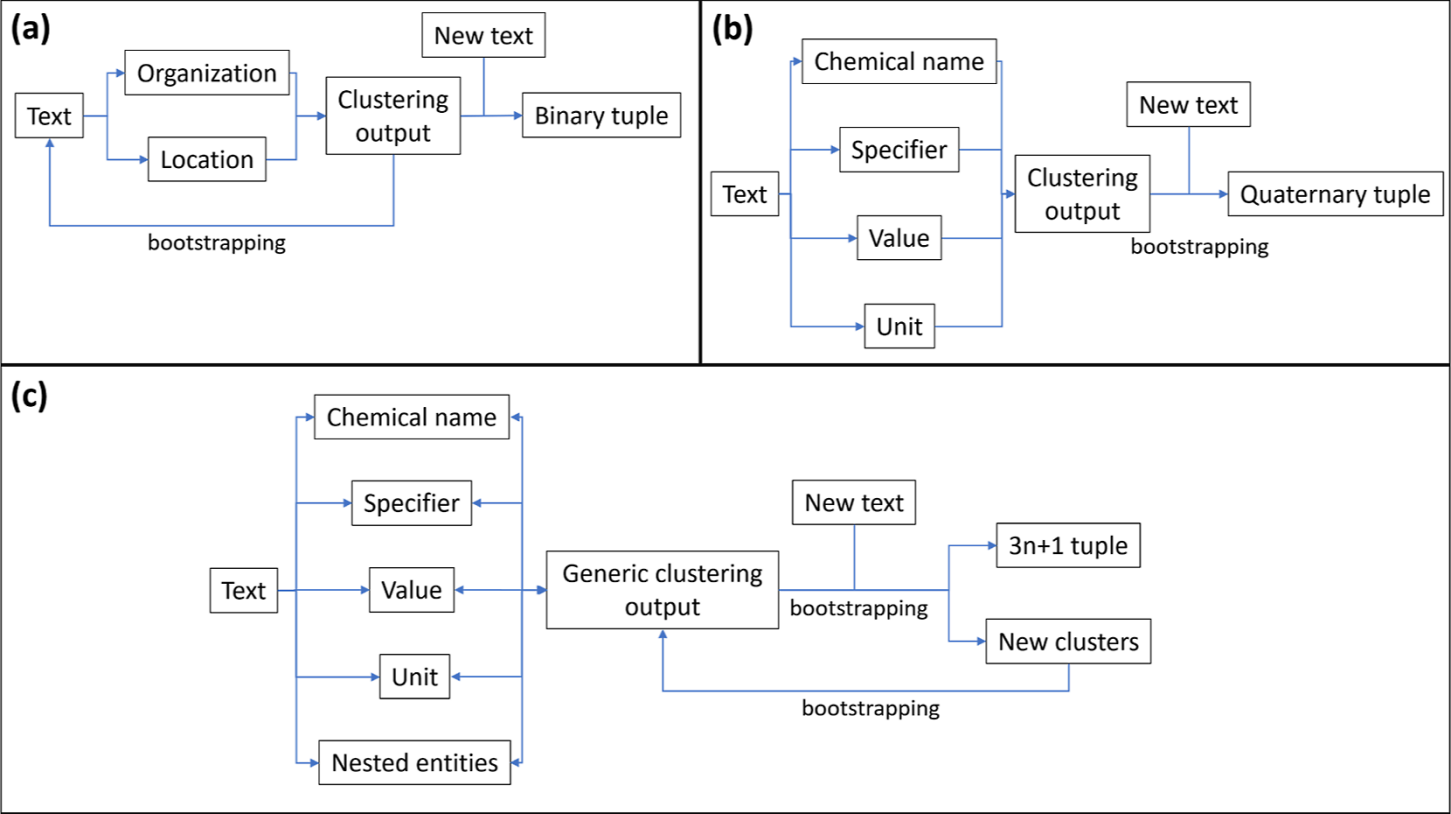
Structures of original Snowball algorithm (a), Snowball 1.0 (b), and Snowball 2.0 (c). Boxes contain textual data; single-ended arrows indicate computing processess where the tail box provides input for the head box; and double-ended arrows indicate reversibility in the clustering stage.

One such software toolkit, ChemDataExtractor,^10−12^ is a “chemistry-aware” data-extraction pipeline that can automatically generate databases of material properties from scientific documents.^16−22^ The Snowball parser is one of the sentence parsers that have been incorporated into ChemDataExtractor 2.0. Originally developed for the extraction of binary tuples {entity 1, entity 2} from newspaper documents^23^ (Figure 1a), the Snowball algorithm was modified by Court and Cole^16^ to extract chemical information on materials from scientific papers, and output data records as quaternary relationships of the form {chemical name, property, value, unit}(Figure 1b). It is based on a semisupervised machine-learning model that can assign a confidence score to each data record to represent the likelihood of it being correct; its algorithm also has bootstrapping capabilities in that it can learn from new information to boost its performance over time. It has been reported^24^ that the Snowball parser can achieve a performance of around 90% and 50% for precision and recall, respectively, which defines its status as a high-precision, low-recall parser.

Despite its performance capabilities, the Snowball parser has demonstrated some limitations in its practical applications. One limitation is that the output is not flexible enough, resulting in the parser not being able to realistically achieve a higher recall at lower precision. Another is the training process being property-specific, such that whenever the Snowball parser is migrated to new chemical properties, a new set of labeled sentences must be provided to train the model, which requires considerable time and effort from the user.

In this work, we present Snowball 2.0: an improved sentence-level parser for chemical data extraction using ChemDataExtractor. Compared with its predecessor, Snowball 2.0 has been designed with several new benefits: (1) generic data extraction, whereby information about different material properties can be extracted by one Snowball model, thus eliminating the requirement for manual training; (2) better performance, whereby a Snowball model can achieve higher recall without compromising precision; (3) better bootstrapping, whereby a Snowball model can learn from new information faster and more efficiently; (4) improved model stability, user-friendliness, and support for nested models have been realized by various changes to the algorithm. See Figure 1c for a visual representation of the structure of Snowball 2.0.

In the following sections, we will describe how Snowball 2.0 can be used in conjunction with ChemDataExtractor,^12^ discuss how its new methodologies function during training and data extraction processes, highlight its key features and any differences from Snowball 1.0, and finally evaluate its performance by training a Snowball model and optimizing the hyperparameters, thus demonstrating its performance improvements over the previous version.

## Implementation

### Workflow

**Figure 2:**
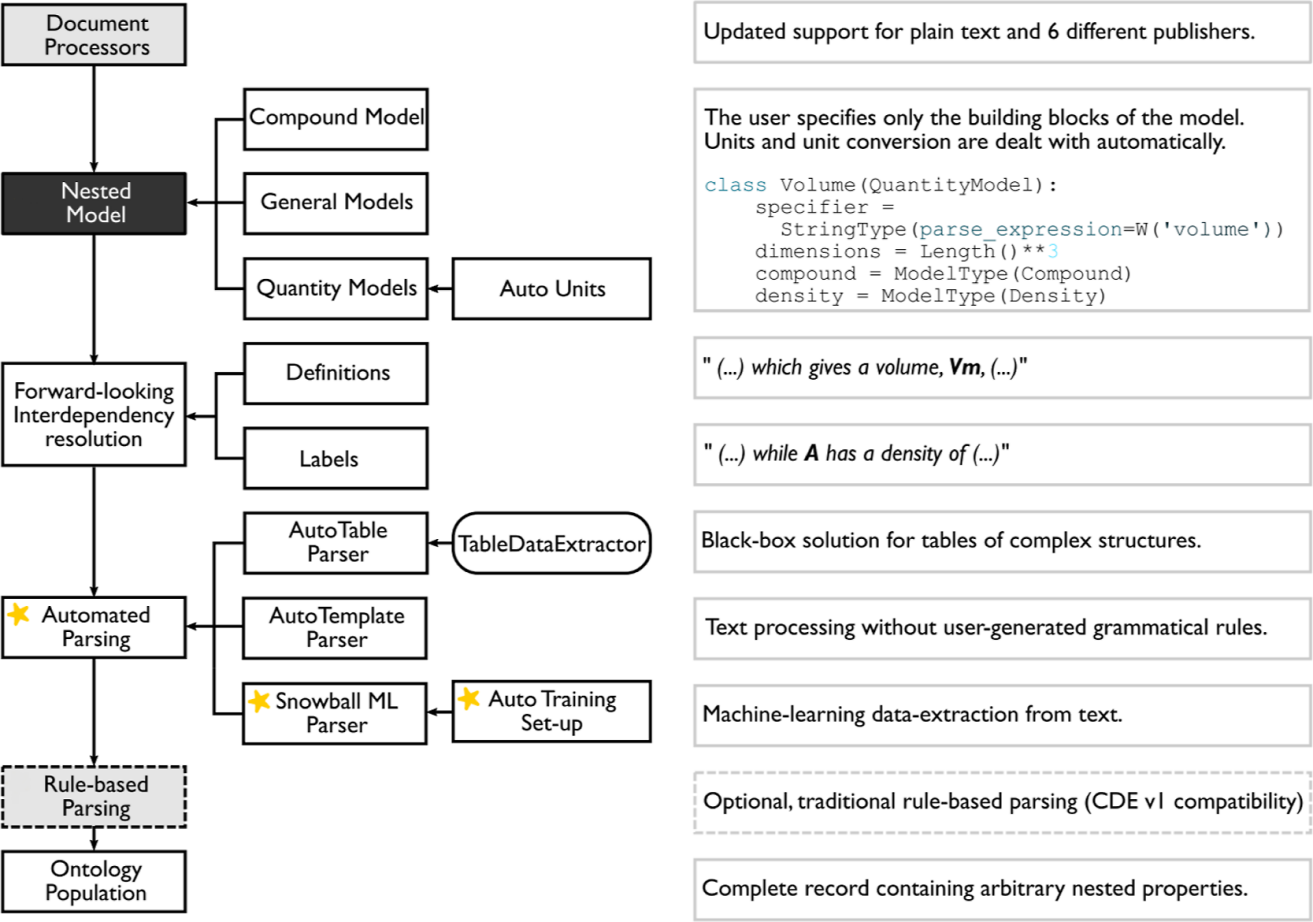
Complete pipeline of chemical data extraction using ChemDataExtractor 2.0. Yellow stars indicate necessary user interaction, and the boxes with dashed borders are optional steps. Adapted with permission from ref (11). Copyright 2021 American Chemical Society.

Snowball 2.0 is a sentence-level parser implemented in the phrase parsing stage of the ChemDataExtractor pipeline for automated information extraction from text. Figure 2 shows how this parser is integrated into the overarching pipeline, which is now described to provide a general background. First, the document processor converts document files of various formats into document objects based on the hierarchical structural information on scientific articles, such as the title, abstract, paragraphs, tables, and figures, which are embedded in the markup language of HTML and XML files. The next stage, forward-looking interdependency resolution, consists of a series of steps including sentence splitting, tokenization, part-of-speech tagging, and entity recognition so that sentences are represented as a stream of word tokens, each tagged by their syntactic function. Based on the property model defined by the user, certain word tokens can be recognized as property entities, which comprise part of the foundations of chemical relationships for records. A typical property-model definition includes a specifier with its variations, a dimension or unit, a compound, and optional nested properties. The final stage, automated phrase parsing where Snowball 2.0 is positioned, links individual property entities together to form chemical relationships, from which chemical data can be extracted.

### Comparison with Rule-Based Parsers

Unlike the AutoTemplateParser, which extracts chemical information by matching sentences to prewritten grammatical rules, the Snowball ML parser compares sentences with labeled phrases based on textual similarity using the bag-of-words model. Accordingly, the Snowball parser offers several benefits compared with traditional rule-based parsers. The most apparent one is a confidence estimate that is made on individual data records and can be calculated from the textual similarity metric. In contrast, AutoTemplateParser, like most other rule-based parsers, has deterministic outputs: the process of extracting data records from text either succeeds or fails such that the overall quality of the database can be evaluated by manual examination but the correctness of individual data entries cannot. Also, the confidence scores from the Snowball parser allow for databases of different characteristics to be generated from one data extraction process. For example, by simply setting a minimum confidence value, data records with low confidence can be excluded from the output, resulting in a smaller and cleaner database.

## Training

### Terminology

Here, we lay out the naming conventions in the Snowball parser. A summary of the definitions is shown in Table 1. For a sentence, each compound can have only one chemical relationship (data record), and the word tokens that make up a chemical relationship are the entities, which are tagged by ChemDataExtractor before they are passed to the Snowball parser. Snowball 2.0 has better support for nested property models than its previous version, such that the number of entities in one chemical record can be 3*n* + 1, where *n* is the number of property models, three comes from the specifier-value-unit combination, and the last single entity refers to the compound that is described by the *n* property models. Entities divide a sentence into multiple segments, and those segments of word tokens are termed elements, each of which can be a prefix, middle, or suffix, depending on its relative position from an entity. To discard unnecessary information, prefix and suffix elements have a limited number of word tokens and their lengths can be adjusted by the user. Also, each element has adjustable weight, so that certain parts of a sentence can have higher importance in the calculation of the confidence score. Entities together with elements form the basis of a phrase, which is essentially a labeled sentence, and each phrase carries a confidence score, which describes the probability of it being correctly labeled.

**Table 1:** Descriptions and Examples of Snowball Objects

object name	description and example
sentence	sentence split from the body text. “The bulk TiO_2_ has a direct band gap of 3.2 eV at tau point.”
entity	tokens that correspond to either a compound, a specifier, a value, or a unit. “Band gap”
chemical relationship	a list of entities (compound, specifier, value, and unit) that resembles a complete chemical record. [TiO_2_, band gap, 3.2, eV]
prefix	word tokens before the first entity. [“The”, “bulk”]
middle	word tokens between each relationship entity. [“has”, “a”, “direct”, “of”]
suffix	word tokens after the last entity. [“at”, “tau”, “point”]
weight	normalized importance factor for the prefix, middles, and suffix. (0.1, 0.8, 0.1)
phrase	a combination of entities and elements with a confidence score. [“The bulk”, chemical name, “has”, “a”, “direct”, specifier, “of”, value, unit, “at tau point”]

### Relationship-Based Training

As a semisupervised machine-learning method, the first step is to train the Snowball parser in a supervised manner. A set of labeled data, or phrases, must be curated for training. This is achieved by manually annotating the chemical relationships in the training sentences. In the field of natural sciences, sentences usually contain no more than a few entities and only one chemical relationship. Therefore, the Snowball algorithm generates all possible combinations of entities to form a list of candidate relationships, and the user identifies the correct candidate relationships from the list.

### Entity-Based Training

Unfortunately, there are cases where dozens of entities and multiple chemical relationships can be found in a sentence so that the number of candidate relationships can easily exceed several hundreds. Training these sentences requires a significant amount of effort from a user, and the possibility of human error becomes nontrivial. Therefore, we added entity-based training into Snowball 2.0: when the number of candidate relationships in a sentence exceeds a threshold value, which can manually be adjusted by the user, the algorithm switches to entity-based training. The user chooses one correct entity out of all candidate entities of a given category; the algorithm is then rerun for each category depending on the requirement of the property model; this step is repeated until all relationships have been constructed. In other words, the training process is transformed into multiple single-choice problems rather than one multiple-choice problem with too many options. In practice, we have found that this approach is more user-friendly and less time-consuming.

## Clustering

### First Level

To improve processing efficiency, all phrases curated during training are assigned to clusters, each of which is a group of similar phrases that can be represented as one. To accommodate different ordering of entities and textual variation, a two-level hierarchy is implemented during the clustering process. The first level separates phrases into several “pseudo” clusters, according to the number and ordering of entities per phrase. Only phrases from the same cluster are comparable in terms of similarity.

A clustering procedure was administrated in the previous revision of the Snowball algorithm. In Snowball 2.0, the first-level clustering has been redesigned in order to realize generic property models, such that phrases based on different property models are also comparable. This redesign involved localizing the comparison of entities to individual property levels rather than on the whole property-model level. For example, phrase 1 “At 300 K, TiO_2_ has a band gap of 3.2 eV.” can be described by two property models: *Temperature* nested on *BandGap*, and its corresponding entity list reads as [*Temperature*_*value*, *Temperature*_*unit*, *Compound*_*name*, *BandGap*_*specifier*, *BandGap*_*value*, *BandGap*_*unit*]. Phrase 2 “The 100 nm Cobalt quantum dot has a Curie temperature of 1394 K.” belongs to a property model of *Diameter* nested on *Curie Temperature*, and its associated entity list reads as [*Diameter*_*value*, *Diameter*_*unit*, *Compound*_*name*, *Curie*_*specifier*, *Curie*_*value*, *Curie*_*unit*]. For phrase 1, the main property *BandGap* has its entities indexed at positions [3, 4, 5] in the list, and the order goes as [*specifier*, *value*, *unit*]; for the nested property *Temperature*, the indices and order of its entities are defined as [0, 1] and [*value*, *unit*], respectively. For phrase 2, the indices and order for its main and nested properties are identical to those for phrase 1. Therefore, phrases 1 and 2 are comparable and belong to the same pseudocluster. This generalization also allows a Snowball parser to be applied to entirely different property models without any additional training. This, combined with the ability to import and export labeled phrases across Snowball parsers, makes these models much easier and quicker to use.

Snowball 2.0 differs from the previous version in that the clustering process is executed after training rather than simultaneously, and the process is now fully reversible. This transition was proven necessary for the optimization of hyperparameters, as changing their values would alter the structure of clusters, which would require all phrases in the Snowball parser to be retrained in the previous version. Being independent and reversible, the clustering process gives the user the possibility to experiment on hyperparameters without repetitive training, so that the optimal values of hyperparameters can be determined entirely automatically.

### Second Level

The second level of clustering concerns textual similarity between phrases under the same pseudocluster. First, the elements of a phrase (prefixes, middles, and suffixes) are converted into a normalized vector of unit length, where each vectorial direction corresponds to an element. The value for each element is a dictionary of word tokens and their weights, which are calculated using a Term Document Frequency model^25^ that assigns heavier weights to more frequently appearing tokens. Next, the similarity between two phrases *p* and *q* is defined as
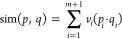
1where *v*_*i*_ is a normalized importance weight vector, *m* is the number of entities, and the sum spans all elements. The first-level clustering ensures that phrases *p* and *q* have the same number of elements, and that (*p*_*i*_·*q*_*i*_) is a dot product of two vector elements, where all unique word tokens in the combined dictionary are treated as a basis set.

Our second-level clustering mechanism builds upon the Single-Pass Classification Algorithm^26^ (SPCA): the first phrase is assigned to a new cluster; the subsequent phrase gets assigned to the same cluster, given that the similarity between the phrase and the extraction pattern of the cluster is equal to or is greater than the minimum similarity threshold, τ_sim_*h*_ (*h* stands for high, whereas the low similarity threshold τ_sim_*l*_ will be introduced in the data extraction section). If none of the similarity scores reach τ_sim_*h*_, then the phrase is assigned to a new cluster. The pseudocode for the second-level clustering process is given in Algorithm 1:
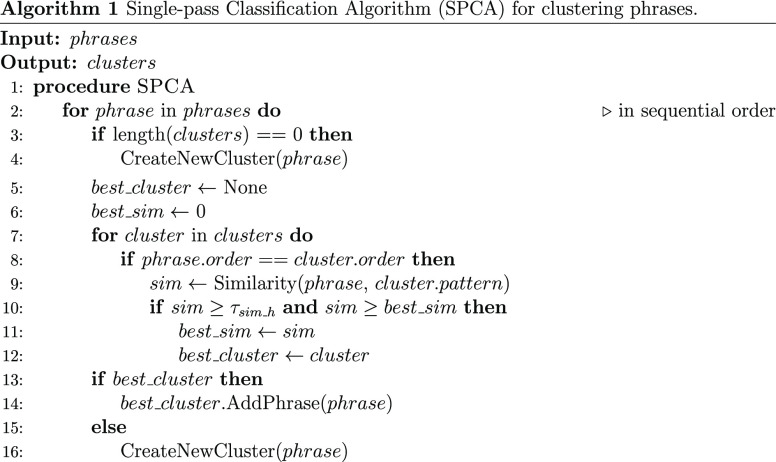


### Extraction Pattern

Once a cluster has been created, all of the included phrases can be represented by one extraction pattern, which itself is a synthesized phrase composed of the most frequent prefixes, middles, and suffixes from all phrases in this cluster. Each extraction pattern has its own confidence score so that an extraction pattern is functionally identical to a phrase.

The original calculation of this confidence score for the extraction patterns has been revised for Snowball 2.0 since we found that the original implementation, which compares the extraction pattern with all phrases within the cluster, can sometimes be unstable. On the one hand, the extraction pattern is a byproduct of phrases, thus by the τ_sim_*h*_ standard, every pair is a positive match. On the other hand, the matching process happens at the training stage; thus, it is not mathematically rigid to run the data-extraction process on those phrases, which relies on confidence scores to distinguish positive and negative matches. Instead, the confidence of an extraction pattern is simply the weighted sum of confidence scores for contributing elements:
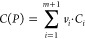
2where *v*_*i*_ are the weights for elements (prefixes, middles, and suffixes), *C*_*i*_ are the confidence scores of phrases whose elements contribute to the extraction pattern, and the sum spans over all elements in the extraction pattern.

### Similarity Triangle

**Figure 3:**
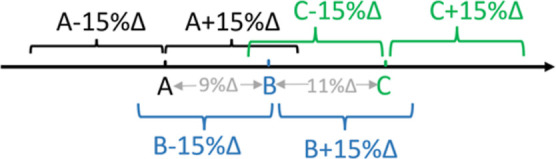
Simplified representation of single-element phrases on a 1D line. Three phrases *A*, *B*, and *C* are shown as points, and curly brackets cover the range of phrases that can be matched to these phrases by employing τ_sim_*h*_.

The motivation behind the implementation of the SPCA was to reduce the number of phrases needed in computation: from the number of all phrases to the number of clusters, thus improving the processing speed. The user can tweak the shape of clusters, both in number and size, by adjusting the value of τ_sim_*h*_. However, the execution of this process, if carried out without modification, brings up two ambiguities: redundant information and the training sequence dependency. The former refers to the possibility that a new phrase could match multiple clusters simultaneously and is repeatedly added to those clusters. This issue has been fixed in Snowball 2.0, and the user is given the option to add new phrases to only the best matching cluster, as shown in Algorithm 1. The latter refers to the fact that the exact shape of the clusters is highly dependent on the ordering or sequence of training phrases. For example, imagine that τ_sim_*h*_ = 85%, we have three phrases *A*, *B*, and *C*, and the similarity scores are sim(*A*, *B*) = 91%, sim(*B*, *C*) = 89%, and sim(*A*, *C*) = 80%, respectively. For the sake of simplicity, we will restrict our analysis to a single element, which projects onto a 1D line, as shown in Figure 3.

In the first case, we consider the sequential order: *A*, *B*, and then *C*. First, *A* is assigned to a new cluster, and the extraction pattern is exactly phrase *A*. Since sim(*A*, *B*) > τ_sim_*h*_, *B* is assigned to the same cluster. The extraction pattern always favors the newest information; thus, it is now dominated by phrase *B*. When *C* is added, the similarity between it and the extraction pattern *B* is higher than τ_sim_*h*_, therefore *C* is also added to the same cluster, and one single cluster is generated.

In the next case, we consider a different order: *A*, *C*, and then *B*. Similarly, *A* is assigned to a new cluster with the extraction pattern being *A*. Next, *C* is farther away from the phrase *A*; therefore, it is assigned to the second cluster. Finally, *B* can be assigned to the first cluster as it is closer to *A* than *C*. In the end, two clusters are generated, which is different from the first case and will yield different performance. In other words, tightly packed phrases generate fewer clusters than sparsely arranged phrases.
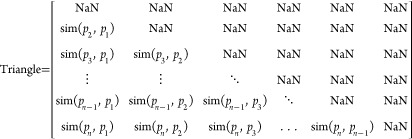
3

To alleviate the clustering problem that is associated with this varying order of phrases, we equipped the SPCA with a more advanced sorting mechanism. A “similarity triangle” method was used to sort the phrases in a way that maximizes the number of clusters; rather than sorting them sequentially. Thereby, the resulting clusters are immutable regardless of the order in which the phrases are trained. The similarity triangle refers to a 2D square matrix, whose diagonal is empty and cuts the matrix into two triangles, whereby calculations are administered on only the element of one of these triangles. The side length of the isosceles triangle is the number of phrases under a pseudocluster minus one, and each point on the triangle represents the level of similarity between the two phrases from the two sides. From the previous example, clustering in pairs of phrases with the lowest similarity gives the highest number of clusters and vice versa. Therefore, the steps are as follows: find the minimum similarity score in the triangle, locate the corresponding two phrases, cluster them using SPCA, and delete the corresponding row and column from the triangle; repeat this step until the triangle is fully cleared. This mechanism ensures that all phrases are clustered in the same order regardless of how they appear in the training pool and that the resulting clusters can have more consistent performance. Algorithm 2 summarizes this method.
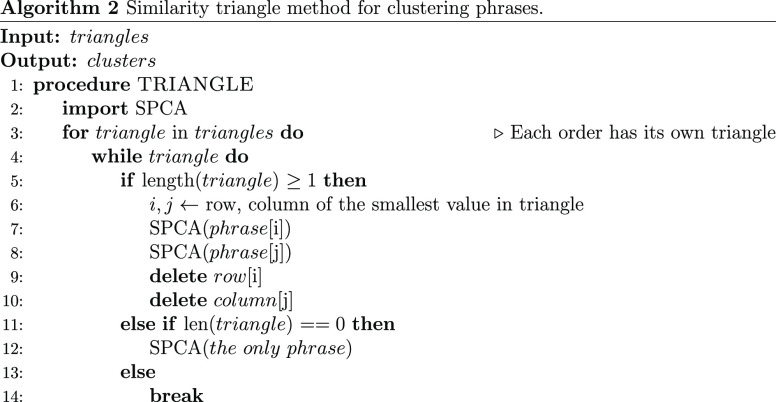


## Data Extraction

### Overview

Once a set of training sentences has been labeled, the Snowball parser can extract chemical information from new sentences via unsupervised learning. The first step is to find all candidate chemical relationships that correspond to the property model, exactly as the first step in relationship-based training. Then, different combinations of chemical relationships form different candidate phrases, which are compared to existing clusters. The one candidate phrase with the highest likelihood of being correct, or the highest confidence score, is chosen, from which new information can be extracted.

### Learning Rate

One of the defining features of the Snowball parser is its ability to learn and refine itself from new information: certain candidate phrases with sufficiently high confidence scores can be added to the clusters. The extraction patterns are also updated to reflect this new information, so that the next time the same sentence is presented to the Snowball parser, the best candidate phrase will be more similar to the extraction patterns and will receive a higher confidence score, such that it is more likely to be accepted. This effect can also be described as a bootstrap, whereby the Snowball parser continually learns from previously unseen sentences and favors information that is more correct and frequent, thus improving its performance over time.

However, as partially highlighted in Dong and Cole,^24^ Snowball 1.0 may suppress active learning to some extent. The Snowball model updates the extraction patterns and their confidences *C*(*P*) at a certain pace, or at a learning rate α, which is defined through
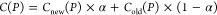
4so that the confidence scores of extraction patterns can be updated with a “delay”. Setting this learning rate to 1 means the extraction patterns and the confidence scores are always synchronized, whereas a learning rate of 0 means total asynchronization, and the confidence scores are fixed, though the extraction patterns are still updated in real time. It was previously found that the act of leaving the predefined learning rate at 1 during data extraction would yield poor recall; this is a direct consequence of lowering the confidence scores of extraction patterns by unconditionally adding unknown-quality phrases into the clusters and diluting the quality of labeled phrases that had been curated during training. Setting the learning rate to 0 would partially alleviate this problem but would also cut the connection between the extraction patterns and their confidence scores, in which case the Snowball model cannot truly learn.

In Snowball 2.0, the hyperparameter learning rate is depreciated so that it is permanently set to 1 without the downsides of suppressed recall. This was achieved by revising the confidence calculations for extraction patterns and new relationships to better reflect the correctness of new information and by employing an extended data-extraction process so that the clusters can be updated in a controllable manner. Additionally, a Snowball model can further learn from new information by generating new clusters that are composed fully of extracted phrases that have sufficiently high confidence scores but are not sufficiently similar to any of the extraction patterns. This process will be discussed in detail in later sections.

### Extended Similarity

It was previously found that the τ_sim_*h*_ value can have a significant impact on the performance of a Snowball model, and the optimal τ_sim_*h*_ value for data extraction is usually smaller than the preferred value for use in training. Since lowering the τ_sim_*h*_ value allows more matches to be made between a candidate phrase and the extraction patterns, candidate phrases are more likely to be accepted, which greatly improves recall at the cost of slightly reduced precision. However, changing the τ_sim_*h*_ value for the data-extraction task would lead to a difficult choice between sacrificing performance and breaking the sorting standard of clusters. Therefore, it is necessary to isolate the function of τ_sim_*h*_ during the data-extraction process and assign it to a separate hyperparameter, the low similarity threshold τ_sim_*l*_, which is less or equal to τ_sim_*h*_. Consequently, when evaluating a candidate phrase, all extraction patterns that have similarity scores higher than τ_sim_*l*_ are considered to be good matches.

**Figure 4:**
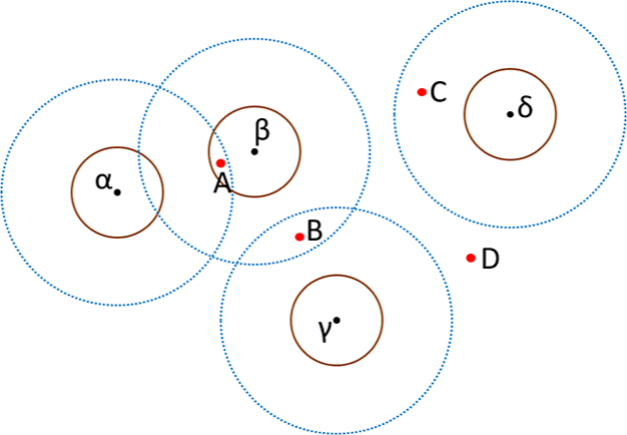
Illustration of the extended similarity metric with examples. The extraction patterns of four clusters α, β, γ, and δ are shown as black dots, and the coverage of τ_sim_*h*_ and τ_sim_*l*_ are shown as brown and blue circles. Note that solid and dashed circles can overlap, as the clustering process does not consider the effect of τ_sim_*l*_. Candidate phrase *A* is compared with cluster β only; candidate phrase *B* is compared with clusters β and γ; candidate phrase *C* is compared with cluster δ; and candidate phrase *D* does not match any clusters.

So far, the similarity metric can be described by three segments: [τ_sim_*h*_, 100%] for a very good match; [τ_sim_*l*_, τ_sim_*h*_) for a fairly good match; and [0%, τ_sim_*l*_) for a poor match. In the process of data extraction, τ_sim_*h*_ and τ_sim_*l*_ describe a hard (exclusive) boundary and a soft (inclusive) boundary, respectively. An example is illustrated in Figure 4 where we consider a simplified 2D plane. An extraction pattern can be drawn as a point, and the corresponding cluster is represented as a solid circle. The area of the circle accounts for the textual variations within the cluster, which is inversely related to τ_sim_*h*_. Therefore, the radius of the circle is also inversely related to τ_sim_*h*_. Similarly, τ_sim_*l*_ defines a larger dashed circle centered on the extraction pattern. When extracting data from a test sentence, any candidate phrase can also be shown as a point. If the point lies inside a solid circle, the candidate phrase is compared to this cluster only, whether it is enclosed by other dashed circles or not. In this case, the solid circle is exclusive, so that all weak matches are neglected when a strong match is found. If the point lies outside any solid circle and inside one or more dashed circles, the candidate phrase is compared to all relevant clusters. In this case, the dashed circles are inclusive, and all relevant clusters are equally considered. Finally, if the point lies outside any dashed circle, the candidate phrase is considered out of range and will be discarded.

### Confidence Calculation

The confidence score *C*(*r*_c_) of a candidate relationship, *r*_c_, derived from a candidate phrase, *p*_c_, is defined as
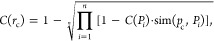
5where *i* corresponds to all clusters of the same order, including the newly generated ones, *P*_*i*_ are the extraction patterns, and *C*(*P*_*i*_) are their confidence scores. This definition differs from Snowball 1.0 in that the product of terms is now normalized by the number of terms, so that *C*(*r*_c_) is less susceptible to other hyperparameters such as τ_sim_*h*_, which affects the number of clusters and thus the scaling of confidence scores. Similarly, we can also define a minimum confidence threshold, τ_c_, to describe whether or not a candidate phrase has a sufficiently high likelihood of being correct for cluster generation.

**Figure 5:**
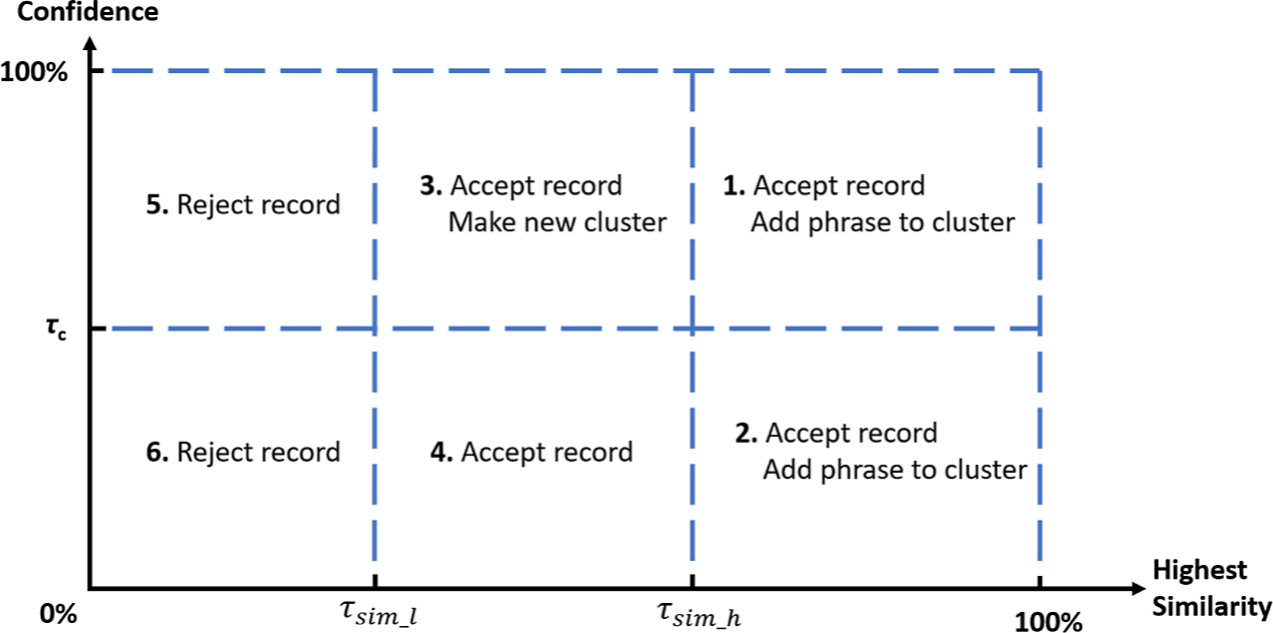
Illustration of how the data-extraction process handles different phrases in Snowball 2.0 according to their confidence scores and highest similarity scores.

With the extended similarity metric and the confidence metric, we can define a complete data-extraction process that is described in algorithm 3. For a new sentence, the model first finds all valid combinations of entities to generate a list of candidate phrases. Each candidate phrase can be divided into six categories according to its confidence score and its highest level of similarity, as shown in Figure 5. In regions 1 and 2 where the highest similarity score of the candidate phrase is greater than τ_sim_*h*_, the phrase is accepted and added to the cluster of highest similarity. As the effect of other extraction patterns is ignored, the candidate phrase is compared with the internal phrases of this cluster, and the confidence score *C*(*r*_c_) can be rewritten as
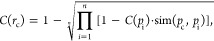
6where *i* multiplies over all phrases *p*_*i*_ within the cluster. In regions 3 and 4, the highest similarity score is between τ_sim_*l*_ and τ_sim_*h*_, so the candidate phrase is accepted; when the confidence score is above τ_*c*_, the candidate phrase is considered sufficiently correct but not sufficiently similar to any of the extraction patterns; therefore, it is assigned to a new cluster, which will boost the confidence of similar new phrases. In regions 5 and 6 where the highest similarity score is below τ_sim_*l*_, the candidate phrase always has zero confidence and is therefore rejected. Finally, if a candidate phrase is accepted, then all data records associated with the phrase can be channeled back into the ChemDataExtractor pipeline for further processing.
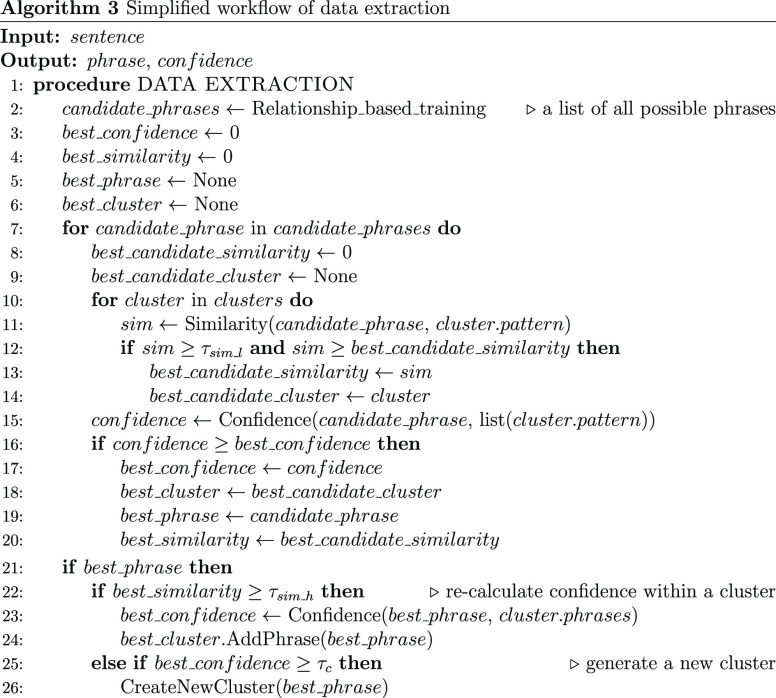


## Evaluation

### Overview

In this section, we will evaluate the performance of the Snowball parser, analyze how it behaves with respect to the hyperparameters, and compare it with the performance of the previous version of Snowball. The performance of the parser was quantified based on the metrics of precision, recall, and *F*-score, which are defined as
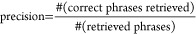
7
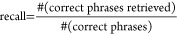
8
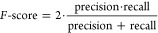
9where precision and recall, respectively, describe the accuracy and coverage of the retrieved data, and *F*-score is the harmonic mean of precision and recall.

In this work, the precision, recall, and *F*-score correspond to the performance of the Snowball parser alone instead of the performance of the ChemDataExtractor pipeline. This is different from previous works^16,24^ related to Snowball 1.0, where errors (false positives and false negatives) that had been introduced during Chemical Named Entity Recognition were also reflected in the performance values. By manually correcting all wrongly identified chemical entities, we can remove input bias that may negatively affect the hyperparameter tuning process. Also, the validation of the Snowball parser was fully automated to eliminate any potential human error when counting the data records. To facilitate this automation, the testing set of sentences has been prelabeled via the training process, and an extracted phrase needs to be 100% matched to the test phrase to be counted as a true positive. Such a standard is much more demanding relative to manual validation, where minor incorrectness is usually ignored (for example, where only one out of two specifiers has been extracted). As such, the performance values shown in this work are not directly comparable to most other works related to ChemDataExtractor.^16−22,24^ To make the comparison between the performance of the Snowball parser and the performance of its previous version^24^ as close as possible, we chose the same property model for training and testing, namely, the band gap property for semiconductors. Around 1500 sentences were randomly selected from related articles published on Elsevier, Springer, and the Royal Society of Chemistry, and they were manually labeled for training and testing. The training and testing sets were split with a 5:1 ratio. The performance of Snowball 1.0 has also been recalculated under the current protocol; see section Point-by-Point comparison with Snowball 1.0.

### Performance Scaling

**Figure 6:**
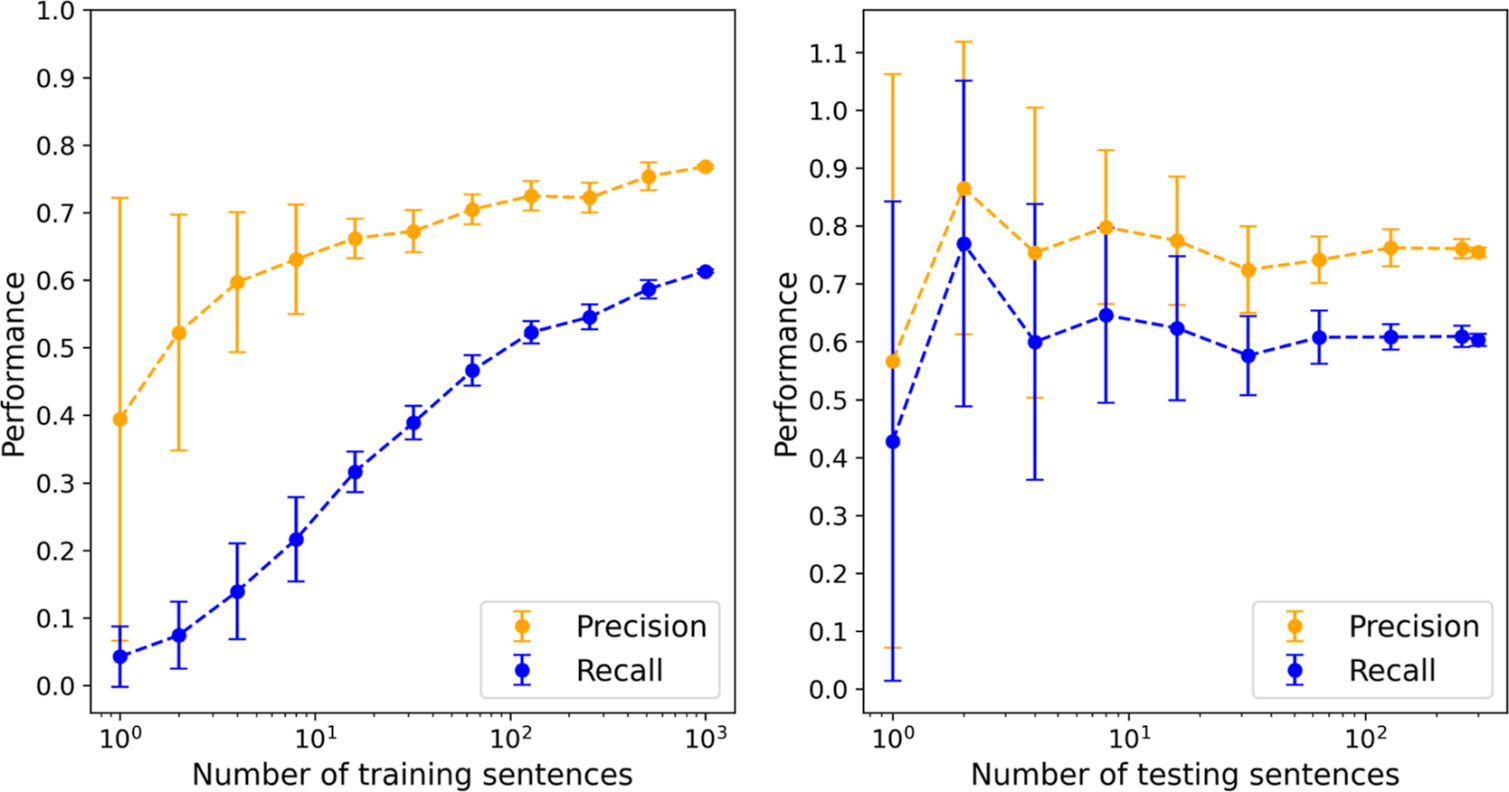
Performance scaling for Snowball 2.0 with the size of the training set (left) and stability scaling with the size of the evaluation set (right). Both *x*-axes are plotted in logarithmic scale to better demonstrate convergence. Data values and their errors are calculated from the mean and standard deviation of 30 repeated tests with randomly selected samples.

Before evaluating the hyperparameter tuning stage of this Snowball model, it is necessary to establish performance and stability scaling with the size of training and testing data, as shown in Figure 6. For the training set, both precision and recall increase with the number of labeled phrases, where the increment between steps drops exponentially. This is expected as more labeled data would always yield better performance. With more than 1000 labeled phrases, the standard deviations are both less than 1%, and a further 5% increase in performance would require at least three times more training data. Therefore, we concluded that the training set is large enough. For the testing set, both precision and recall exponentially converge to steady values, whereas the standard deviations are at 1% with over 300 testing sentences, indicating that the size of the testing set can yield sufficiently stable results.

### Hyperparameter Tuning

**Figure 7:**
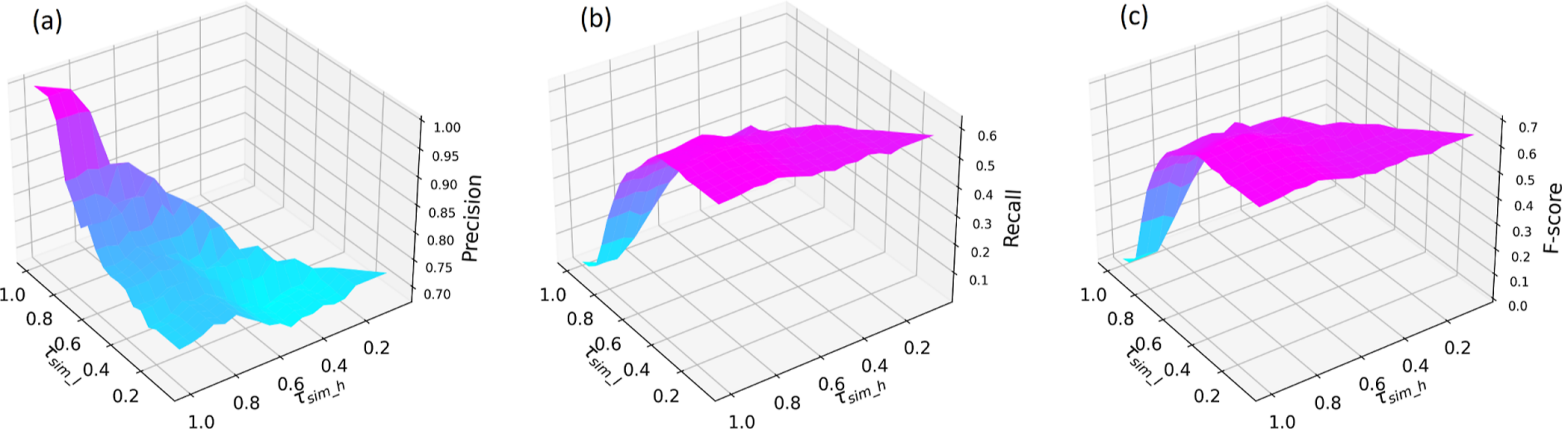
Precision (a), recall (b), and *F*-score (c) for Snowball 2.0 with respect to the high confidence threshold τ_sim_*h*_ and low confidence threshold τ_sim_*l*_. By definition, τ_sim_*h*_ ≥ τ_sim_*l*_, hence the input parameter grid is a triangle rather than a square. All three plots are set to the same viewing angle.

Since the confidence threshold τ_c_ only governs cluster generation from unseen sentences, which has little performance impact given the small size of the evaluation set relative to a database, here we will focus on maximizing precision, recall, and *F*-score by optimizing the two similarity thresholds τ_sim_*h*_ and τ_sim_*l*_. The results are shown in Figure 7, where the performance can be described by a point on a surface in a 3D parameter space.

For precision, maximum values are achieved at around τ_sim_*h*_ = 0.8 for all τ_sim_*l*_ values (Figure 7a). This is unlike the previous version of Snowball^24^ which favors having τ_sim_*h*_ ≈ 1.0 to maximize the diversity in its extraction patterns. For the recall, optimal values for Snowball 2.0 are found at around τ_sim_*h*_ = 0.8; it monotonically increases with decreasing τ_sim_*l*_ and gradually flattens out at around τ_sim_*l*_ = 0.2 (Figure 7b). In contrast, recall in Snowball 1.0 reaches a plateau as early as τ_sim_*l*_ = 0.6 and can sometimes drop by lowering τ_sim_*l*_.^24^ Finally, the behavior of the *F*-score for Snowball 2.0 (Figure 7c) closely resembles the surface projection of recall in Figure 7b, where the maximum value is at around τ_sim_*h*_ = 0.8 and flattens out after τ_sim_*l*_ = 0.4. For comparison, Snowball 1.0 showed a clear peak *F*-score at τ_sim_*l*_ = 0.65; at lower τ_sim_*l*_ values, the precision deteriorates quickly and the average performance is reduced.^24^

**Figure 8:**
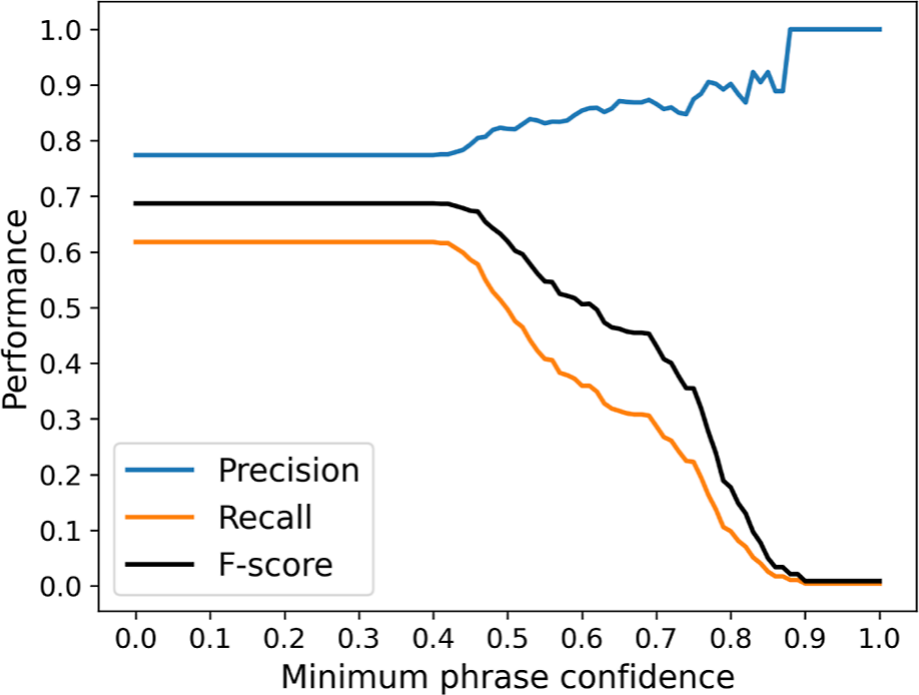
Performance of Snowball 2.0 when implementing a minimum phrase confidence filter. τ_sim_*h*_ and τ_sim_*l*_ are set to 0.75 and 0.4, respectively, in this configuration.

In terms of the confidence score, it was previously found that the implementation of a minimum phrase confidence filter in Snowball 1.0 when compiling phrases into a database would negatively affect the performance. This statement still holds for Snowball 2.0, as shown in Figure 8, where the recall and *F*-score reach maximum values when the minimum phrase confidence is set to 0%. However, at nonzero values, this confidence filter can successfully remove some false positives to improve precision to nearly 100%, though at the cost of significantly reduced recall. This also suggests that the confidence threshold τ_*c*_ can be set to above 0.8 when the model has >90% accuracy, to ensure that newly generated clusters are very likely to be correct.

Overall, the lower similarity threshold τ_sim_*l*_ has the greatest impact on performance, which is consistent with Snowball 1.0. For optimal *F*-score, we recommend setting τ_c_ to above 0.8, τ_sim_*h*_ to between 0.7 and 0.8, and then adjusting the value of τ_sim_*l*_ between 0.4 and 0.8 to balance precision and recall depending on the performance target.

### Point-by-Point Comparison with Snowball 1.0

**Figure 9:**
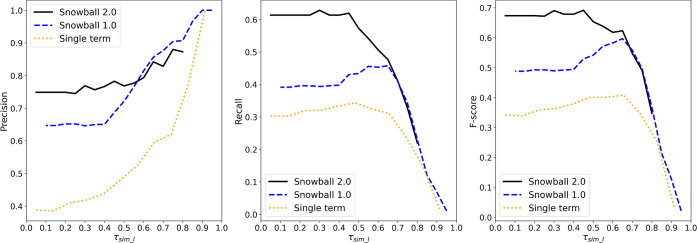
Performance comparison between Snowball 2.0 and Snowball 1.0 in terms of precision (left), recall (middle), and *F*-score (right) as a function of τ_sim_*l*_. All evaluations are cut off at τ_sim_*l*_ = τ_sim_*h*_. Results of the “single term” formulation, a modified eq 5, are also shown for reference.

To better visualize a point-to-point comparison with Snowball 1.0, its performance behavior was simulated by setting both τ_*c*_ and the normalization coefficient *n* in eq 5 to 1. τ_sim_h_ was set to 0.95 and 0.75, respectively, for Snowball 1.0 and 2.0, to represent the best case scenario, while other parameters were kept identical. The results are shown in Figure 9.

In terms of precision, Snowball 2.0 shows less dependency on τ_sim_*l*_ but more fluctuation. This is most likely caused by the normalization of the phrase confidence score that favors good matches on average instead of more matches. As τ_sim_l_ decreases, more distant matching clusters are included in the equation, lowering the normalized confidence score, which locally shifts the ranking of candidate phrases and globally manifests as instability. Above τ_sim_*l*_ = 0.6, Snowball 2.0 performs slightly worse than Snowball 1.0 by a few percent but noticeably better by more than 10% at lower τ_sim_*l*_. For both recall and *F*-score, Snowball 2.0 constantly performs equally well or better than Snowball 1.0 by up to 20%, depending on the value of τ_sim_*l*_. Therefore, we concluded that Snowball 2.0 shows a better performance in most configurations. The only drawback is that the precision cannot go beyond 90% without severely compromising the recall due to the restriction of τ_sim_*l*_ ≤ τ_sim_*h*_; thus, a Snowball 1.0 “emulation mode” has been built into Snowball 2.0, should the user choose to prioritize precision over recall.

We also need to emphasize that the formulation of the phrase confidence score in eq 5 can significantly affect the performance behavior. Equation 5 was proposed based on empirical observations and tested to yield measurable improvements, though at the cost of less flexibility and stability for precision. There are many unexplored ways of defining the phrase confidence score that may yield better or worse results. As a counterexample, given that a candidate phrase is likely to be closest to just one cluster, one may be persuaded to keep only the one best term in eq 5. The result of this formulation, which is also shown in Figure 9 as a “single term” model, proved to be inferior in all configurations. It also implicitly suggests the necessity of parallel clusters, which were initially introduced for the sole purpose of reducing the processing time.

## Conclusions

The increasing popularity of text mining in the field of data-driven materials discovery has necessitated better sentence-parsing methods. The Snowball parser is a sentence-level parser built for the software toolkit, ChemDataExtractor, for the automated extraction of chemical data from the scientific literature. It is based on a semisupervised machine-learning algorithm that can assign confidence scores to extracted data records and dynamically balance its precision and recall. In this work, we present Snowball 2.0: a considerably improved version of the Snowball parser that addresses some of the functional limitations of its previous version and additionally provides better generalizability, performance, model stability, and user-friendliness.

Various changes were made in both the supervised training process and the unsupervised data-extraction process. In the training stage, entity-based training has been added to tackle complex and long sentences, and so, nested property models are now better supported. In the clustering stage, by changing the scope of comparison between phrases, a Snowball model can be applied to any property model without additional training, further reducing the need for user interaction. Also, a similarity triangle sorting method was added for better model invariance against training sentences. In the final stage, the data-extraction pipeline was extended to allow the model to learn from new information more efficiently.

Performance of Snowball 2.0 was evaluated by the metrics of precision, recall, and *F*-score. A Snowball model was trained with semiconductor band gap data and tested against randomly selected sentences in scientific papers. In comparison with Snowball 1.0, the recall for Snowball 2.0 can be enhanced by 15–20% while precision is lowered by only a few percent, resulting in better *F*-scores in nearly all configurations, by up to 20% depending on the similarity threshold. The only downside, which is an inability to achieve ultrahigh precision at low recall, can be overcome by employing a minimum confidence filter or by enabling a Snowball 1.0 emulation mode, which has been built into Snowball 2.0.

The biggest feature of Snowball 2.0 is the removal of per-property-model training. The trained model in this work serves as a basis set of phrases that can be exported to other Snowball models to quickly set up a functioning pipeline and to enhance precision and recall, as shown in Figure 6. Moreover, the drawback of low recall in Snowball 1.0 has also been mitigated. These advantages of Snowball 2.0 would make ChemDataExtractor more capable and more performant in data extraction and autogeneration of material databases.

## Supporting Information Available

Snowball model with over 1000 sentences and list of document object identifiers used for training (ZIP)

## Notes

The source code of Snowball 2.0, as well as a user guide and some examples, can be found at https://github.com/QingyangDong-qd220/Snowball_2.0 and can be freely downloaded under the MIT License. A complete build and full documentation of ChemDataExtractor 2.0 is provided at https://github.com/CambridgeMolecularEngineering/chemdataextractor2 under the MIT License.
